# Source of Explant and Light Spectrum Influence in Adventitious Shoot Regeneration of *Prunus salicina* Lindl. (Japanese plum)

**DOI:** 10.3390/plants14142230

**Published:** 2025-07-18

**Authors:** Carmen López-Sierra, José E. Cos-Terrer, Miriam Romero-Muñoz, Margarita Pérez-Jiménez

**Affiliations:** Department of Plant Biotechnology, Genomics and Breeding, Instituto Murciano de Investigación y Desarrollo Agrario y Alimentario (IMIDA), 30150 Murcia, Spain

**Keywords:** morphogenesis, de novo regeneration, LEDs, 6-benzyladenine, somatic shoots

## Abstract

Light influence on shoot regeneration in *Prunus salicina* is a complex interaction that has been studied for the first time. Japanese plum plants were regenerated from calli and seeds of the scion cultivar ‘Victoria’. The effect of four different light spectra (white, blue, red, and mixed), along with three 6-benzyladenine (BA) concentrations (1, 1.5, and 2 mg L^−1^), was studied in these two sources of explants. Organogenic calli were derived from the base of stem explants of the scion cultivar ‘Victoria’, whereas cotyledons and embryogenic axis slices were used as seed explants. Calli cultured with 2 mg L^−1^ of BA and mixed light or 2.5 mg L^−1^ of BA and control light showed the highest regeneration rates, with no significant differences compared to other treatments. Seed explants exposed to 2.5 mg L^−1^ of BA and red light exhibited significantly higher organogenesis. In comparison, those in 1.5 mg L^−1^ of BA with blue light or 2.5 mg L^−1^ of BA with mixed/control light showed no regeneration. BA concentration did not have a significant effect in the induction of somatic shoots from any explant source. In contrast, a strong interaction between light and BA was noticed. This work presents a protocol that can be applied in transformation and editing research as light spectrum studies continue to advance.

## 1. Introduction

Genetic improvement of woody fruit species has been traditionally limited by factors such as heterozygosity and long juvenile periods. As a result of these factors, the time required for cultivar development is significantly extended, and obtaining cultivars with features that are not available in the germplasm of the species can be unfeasible. Genetic transformation or editing enables more precise and efficient modifications, addressing the challenges that are posed by the complex genomes of woody species and significantly reducing the time needed for classical breeding [1]. Nevertheless, a reliable regeneration protocol is considered the bottleneck to improving fruit trees through genetic engineering [2], as regeneration protocols constitute an intermediate step in the process of genetic engineering. However, the efficiency of genetic engineering largely depends on the choice of explant source, which further complicates the regeneration system.

With regard to genetic modification, adult plant material is considered a better choice than juvenile plant material for use as the initial explant. This is explained by the fact that adult material exhibits the features shown by the cultivar from which it originates.

However, juvenile material, such as cotyledons or hypocotyls, has not been evaluated in the field, resulting in an unknown genotype and phenotype. Despite that, juvenile material has been largely used in *Prunus* due to its augmented plasticity in terms of morphogenesis [3,4,5,6], and, although these genotypes are not initially advantageous for plant breeding, they can be utilized in functional studies.

Due to the high recalcitrance of woody species to plant tissue culture, establishing a protocol for de novo regenerated *Prunus* adult material proves difficult. Traditionally, mature tissues have been considered ideal for propagation, although their recalcitrance to de novo regeneration remains a major technical obstacle. However, some authors have reported a two-step system in which callus is induced in the base of shoot proliferation cultures, which is later exposed to an organogenic medium, where de novo shoots would appear [7,8].

Plant regeneration is affected by a range of external and internal factors: culture media, plant growth regulators, light, carbon source, and agar, amongst others. Traditionally, plant growth regulators have been linked to a morphogenetic response in tissue culture. Cytokinins are responsible for the induction of somatic organs [9], and 6-benzyladenine (BA) is the most widely used plant growth regulator, having been utilized as an organogenic regulator in *Prunus*; it has been used, for example, in sour and sweet cherry [10], plum [5], and peach [7].

Although fluorescent lights have been used in tissue culture rooms for decades, they emit a wide range of wavelengths that are unnecessary and of suboptimal quality for promoting plant growth [11]. Thus, recently, light intensity and spectrum studies have gained relevance in plant tissue culture. In recent years, light-emitting diodes (LEDs) have risen in prominence in growth chambers due to their versatility in terms of wavelength, cool-emitting surface, and ability to control the spectral composition. Some authors have found a relationship between red and blue light ratios on morphogenesis regulation in different species by combining different proportions of both components [12,13]. Despite some preliminary studies, the role of the light spectrum in in vitro morphogenesis remains unclear, and the potential light combinations are endless. Plant photoreceptors are thought to function as activators of the adventitious shoot response. However, the effect of light seems to be specific to the plant species, the developmental stage of the plant, or the composition of the medium [11].

This work aims to elucidate the effect of four different LED light spectrum treatments on the morphogenetic response of *P. salicina* cv. ‘Victoria’ explants, using two sources (calli and seeds) at three different concentrations of BA (1.5, 2, and 2.5 mg L^−1^). The study examines the impact of red, blue, mixed (blue and red), and white light (control light) treatments on adventitious regeneration, with the goal of optimizing a reliable protocol for *P. salicina* regeneration. By varying both the light spectrum and BA concentrations, we aim to determine the most effective combination for improving organogenesis and advancing the development of genetically engineered plums.

## 2. Results

### 2.1. Adventitious Shoot Regeneration from Calli

**Figure 1:**
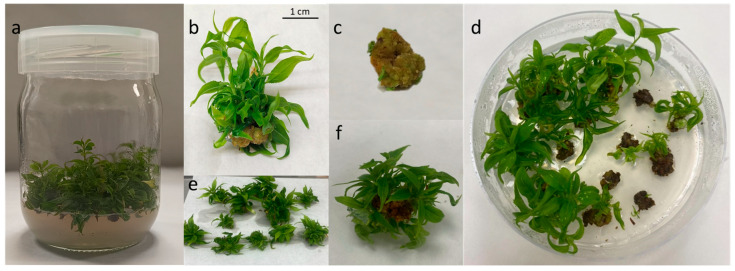
(**a**) ‘Victoria’ clones in multiplication media, (**b**) meristematic bulk, (**c**) callus obtained from the bottom of a meristematic bulk, (**d**) regenerating calli in a Petri dish, (**e**) regenerating calli, (**f**) shoots regenerated from a callus.

After explant preparation and transferring the callus slices to Petri dishes to initiate culture and the de novo regeneration process, the calli turned darker over the course of the culture period. Shoots started to appear (Figure 1d–f) approximately after 10 days of culture, and the calli that induced a higher number of shoots remained green and exhibited increased growth. However, the calli with a lower number of shoots or those that did not show any organogenesis became brown and even died (Figure 1d).

**Figure 2:**
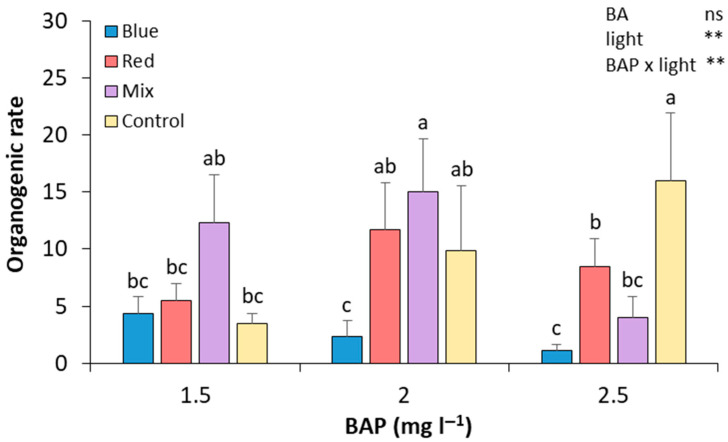
Effect of BA concentration and light spectrum on regeneration rates of calli explants of plum cv. ‘Victoria’. Values are means ± SE. Letters indicate differences (*p* < 0.05) between treatments where *p* ≥ 0.05 means no significant (ns), *p* < 0.01 means very significant (**).

After 28 days of culture in the four different light spectrum treatments and three alternative BA concentrations, the number of shoots was recorded. In control light conditions, the results show that increasing the BA concentration in the culture medium leads to an increase in the regeneration rate (Figure 2). The treatment with 2.5 mg L^−1^ BA under these light conditions showed a significant relative increase in the regeneration rate compared to those calli subcultured in 1.5 mg L^−1^ of BA (by 4.57-fold and 0.61-fold, respectively). The combination of mixed light and 2 mg L^−1^ of BAP produced, along with the control cultured in 2.5 mg L^−1^ of BA medium, the highest regeneration rate, which was significantly higher than that of calli treated with 2.5 mg L^−1^ of BAP under mixed light. Regarding red light, it showed a greater effect on the regeneration rate in those cultivated with 2 mg L^−1^ of BA, compared to 1.5 and 2.5 mg L^−1^ (by 2.10-fold and 1.36-fold, respectively). However, none of the changes was statistically significant. Thus, the calli that attained a higher regeneration rate were those cultured in a media with 2 mg L^−1^ of BA and mixed lights and 2.5 mg L^−1^ of BA and control lights, demonstrating no significant differences with shoots induced in calli cultured in 1.5 mg L^−1^ of BA and mixed lights and in 2 mg L^−1^ and red and control lights (Figure 2). Although the calli cultured in blue lights were the first ones to induce shoots, in the end, these were the calli with the lowest regeneration rates (Figure 2). No statistically significant differences were found between BA treatments. However, differences were found between light treatments, and an interaction between BA and light treatments was also observed (Figure 2).

Table 1 shows the FOC in ‘Victoria’ calli explants. All of the calli that were exposed to red lights in all of the BA concentrations had an organogenic response opposite to that found in the calli grown in the rest of the treatments (Table 1). Compared to control conditions, on average, the relative increases in the FOC in the red light treatment were 20.10%. In contrast, the lowest percentage of organogenic calli was found in the cultures grown in 1.5 and 2 mg L^−1^ of BA under blue light, by significant relative decreases of 1.94-fold and 1.87-fold, respectively (Table 1).

### 2.2. Adventitious Shoot Regeneration from Seed Explants

**Figure 3:**
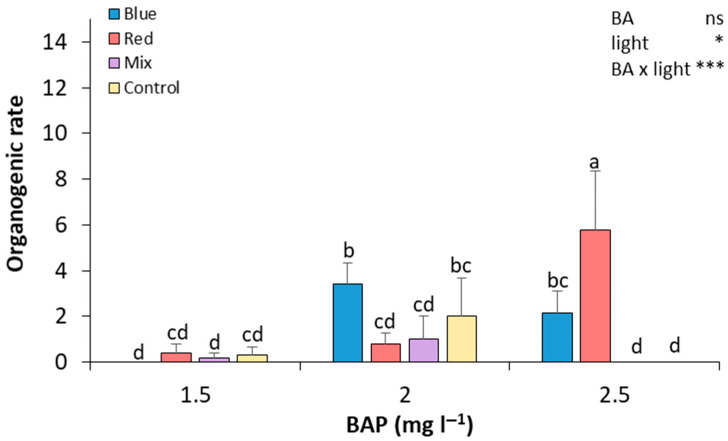
Effect of BA concentration and light spectrum on regeneration rates of seed explants of plum cv. ‘Victoria’. Values are means ± SE. Letters indicate differences (*p* < 0.05) between treatments where *p* ≥ 0.05 means no significant (ns), *p* < 0.05 means significant (*), *p* < 0.001 means very significant (***).

After preparing the seed explants, seed sections were cultured in Petri dishes to start the de novo regeneration process and were evaluated after 28 days of culture. In general, as the concentration of BAP increased, the organogenic rate also increased in most of the light treatments (Figure 3). At 2.5 mg L^−1^ of BAP, red light resulted in the highest organogenic rate, which was significantly higher than in all other treatments, showing a 14.5-fold and 7.25-fold increase compared to explants cultured under red light at 1.5 and 2.0 mg L^−1^ of BAP, respectively. The blue light treatment led to a significant increase in the organogenic rate at 2.0 mg L^−1^ of BAP, with statistically significant differences compared to the red and mixed light treatments under this BAP concentration. At 1.5 mg L^−1^ of BAP, all light conditions resulted in low organogenic rates, with no significant differences between them. Thus, seed explants that were exposed to 2.5 mg L^−1^ of BA and red light showed a higher organogenesis rate, and the difference was significant compared to the rest of the treatments (Figure 3). In contrast, seed explants grown in 1.5 mg L^−1^ of BA and blue light and 2.5 mg L^−1^ of BA and mixed and control light did not show any regeneration. Medium regeneration rates were observed in the rest of the treatments (Figure 3).

**Figure 4:**
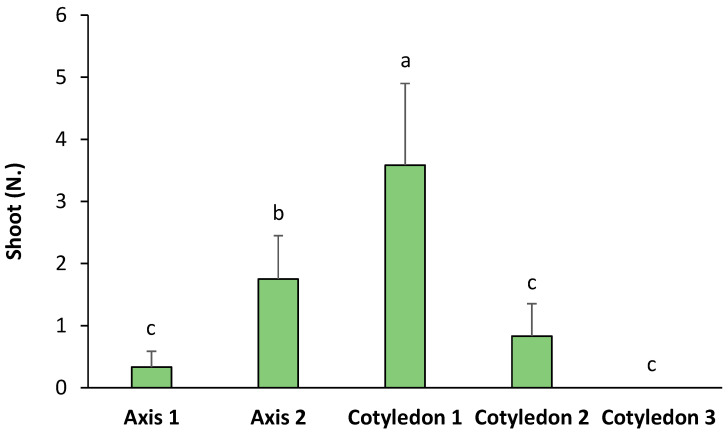
Number of shoots per type of explant in ‘Victoria’ seeds. Values are means ± SE. Letters indicate differences (*p* < 0.05) between treatments.

The origin of the seed explants significantly influenced the number of shoots obtained by de novo organogenesis in ‘Victoria’ (Figure 4). According to the observations, a higher number of shoots were developed from Cotyledon 1 (with a significant relative increase of 2.04-fold with regards to Axis 2, 4.31-fold to Cotyledon 2, and 10.84-fold with regards to Axis 1). Axis 2 also induced a significantly higher number of shoots than the rest of the explants, but lower than Cotyledon 1. The rest of the explants showed no significant differences between them (Figure 4). On the other hand, Table 2 shows the FOSE in ‘Victoria’ seed explants. The results show that red light with 2.5 mg L^−1^, blue light with 2 and 2.5 mg L^−1^ of BA, and the control with 2 mg L^−1^ of BA were the treatments with a higher percentage of responding explants.

**Figure 5:**
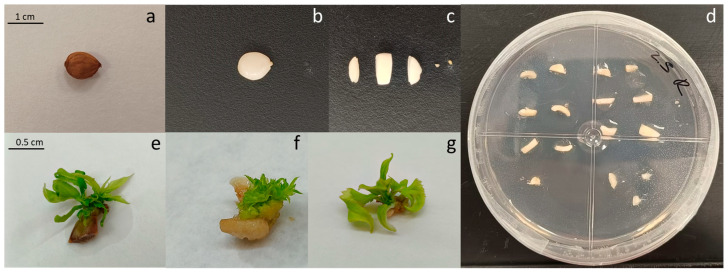
(**a**) ‘Victoria’ seed, (**b**) ‘Victoria’ seed deprived from seed coat, (**c**) seed excision: embryogenic axis and cotyledon slices, (**d**) seed explants in a Petri dish, (**e**) regenerating Cotyledon 1, (**f**) multiple shoots in Cotyledon 1, (**g**) indirect shoot regeneration from embryogenic axis.

**Figure 6:**
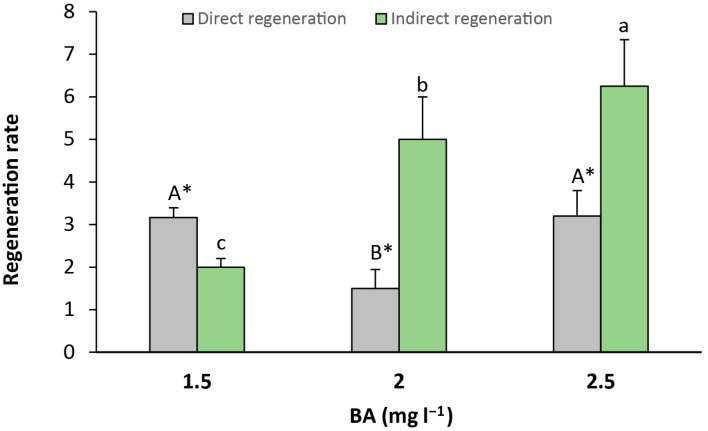
Rate of indirect and indirect regeneration in seed explants per culture media. Values are means ± SE. Letters indicate differences (*p* < 0.05) between BA treatments and asterisk (*) denotes differences between direct and indirect organogenesis.

Finally, direct (Figure 5e,f) and indirect (Figure 5g) regeneration were recorded from seed explants. Direct regeneration was observed in greater amounts than indirect regeneration when the culture media contained 1.5 mg L^−1^ of BA, with a significant relative increase of 1.58-fold in comparison to indirect regeneration. Otherwise, indirect regeneration was significantly higher than direct regeneration in the medium that contained 2 and 2.5 mg L^−1^ of BA, with a relative increase of 3.33-fold and 1.95-fold, respectively (Figure 6).

## 3. Discussion

Advances in regeneration and genetic engineering technologies for *P. salicina* contribute to the genetic enhancement of this species and serve as a valuable tool for molecular research in this crop and other species that are related to it. Compared to other *Prunus* species—and even to the closely related European plum (*P. domestica*)—*P. salicina* remains relatively understudied, which may be linked to the lower level of regeneration of this species [5]. Our results show that juvenile explants are the most suitable for regeneration in *P. salicina*, which aligns with previous studies highlighting the higher plasticity of juvenile explants compared to mature tissues [2,5,14,15]. This finding is crucial, as regeneration in this species is particularly challenging due to its recalcitrant nature, especially when using mature tissues.

Regeneration is often challenging due to the recalcitrant nature of the *Prunus* species, particularly when mature tissues are used. In this report, juvenile and adult materials are tested for their regeneration capacity in different light spectra to improve their proneness to being de novo regenerated.

Even though seed-derived explants have been identified as the preferred source of explants for de novo regeneration in *P. salicina* [2,5,14,15] and other *Prunus* species [6,15,16], in this study, much higher organogenic rates have been attained in calli derived from the base of proliferation clusters (Figure 2) than in seed explants (Figure 3). Likewise, more calli than seed explants (Table 1 and Table 2) responded to the treatments. This would open a new door for genetic engineering in *P. salicina*, since regeneration from adult somatic tissues is highly recommended for clonally propagated crops to maintain the genetic uniformity of the cloned plants, especially for the highly heterozygotic *Prunus* species [17].

The calli produced at the base of the proliferation clusters have been used for adventitious shoot regeneration purposes, yielding outstanding results in recalcitrant species such as grapevine [18] or peach [7,19]. Calli obtained from the base of proliferation clusters have been reported as cells able to continuously regenerate new adventitious shoots [18], which is likely attributable to the prolonged exposure to several cytokinin culture cycles during their formation, a process that could boost the morphogenesis induction process. However, due to the internal balance of hormones, this process is highly dependent on the genotype and must be adjusted not only by species, but also by cultivar [7,19].

As previously explained, the election of the explant is crucial. In terms of seed usage to induce organogenesis, differences between the specific parts of the seed were found. The explants that were closer to the bottom of the embryonic axis (Axis 2 and Cotyledon 1) showed an increased organogenic capacity than those obtained from other parts of the seeds. The study by Canli & Tian [2] used cotyledons for de novo proliferation in *P. salicina*. They observed that the highest regeneration rate was found in the proximal part where the embryonic axis was attached, a part of the cotyledon that would correspond to Cotyledon 1 in this experiment. Hypocotyls have likewise been utilized to induce regeneration in *P. salicina* [5,14] and other *Prunus* species [16,20]. Accordingly, morphogenic capacity in seeds has been reported to be significantly increased when moving from parts that are distal towards parts that are proximal to the hypocotyl [2,21,22]. Hypocotyl explants exhibit dynamic changes in endogenous hormone levels, especially with regard to auxin and cytokinin, favoring shoot regeneration [23]. This spatial and temporal regulation of hormone gradients is crucial for successful de novo organ formation. In *P. serotina* cv. ‘Victoria’, this auxin–cytokinin equilibrium could reach suitability for regeneration in the surroundings of the axis, in the Cotyledon 1 section, where the highest number of shoots was recorded.

Somatic organogenesis is a process where new plant organs grow from non-reproductive (somatic) cells. It can happen in two ways: directly or indirectly. The more common type is indirect, which occurs in three main steps. Firstly, regular plant cells change and form a mass of unspecialized cells called the callus. Then, this callus becomes pluripotent, which means that it gains the ability to turn into different types of plant tissues. Finally, the callus develops into new plantlets. In this experiment, the use of calli as an intermediate step could favor the organogenic capacity of ‘Victoria’ plum seeds. This can also be seen in the results reported by other authors that induced shoots over Japanese plum seeds [2,5], and could be the reason why indirect morphogenesis is more frequent in the literature related to woody plant de novo organogenesis. Based on our results, the indirect pathway of regeneration, involving callus formation, appears to be more suitable for subsequent experiments involving ‘Victoria’ plum seeds. This is supported by the consistent shoot induction observed from the calli in our study and the prevalence of indirect organogenesis reported in the literature for woody species like *Prunus*. Actually, only a few reports show direct de novo regeneration in *Prunus*, and this seems to be more dependent on the hormone endogenous equilibrium of the cultivar than on the plant growth regulators present in the media [6]. Therefore, focusing on indirect regeneration protocols may enhance reproducibility and efficiency in future transformation or propagation studies in this species.

Light conditions have an impact on the in vitro morphogenetic responses of cultured cells and tissues. Our results align with previous attempts to establish a relationship between light spectra and the regulation of morphogenesis [11]; however, there is limited data concerning woody species [24]. In this experiment, light was highly significant in *P. salicina* adventitious shoot regeneration in adult and juvenile explants, whereas no significant results were found with BA alone. This contrasts with the findings of Zielinska et al. [25], who determined that the effect of plant growth regulators was stronger than the influence of light. ‘Victoria’ explants reacted to a combined effect of light and BA. In seed explants, monochromatic lights induced higher regeneration rates, different from those of calli explants, where a broader spectrum promoted regeneration, as previously described by other authors using calli from adult material in woody plants [26,27].

In seed explants, treatments with a broader response in the form of regenerating explants coincided with those with higher regeneration rates. Contrary to the calli, the treatments that obtained a higher regeneration were not the same as those with more explants responding to the treatment. Therefore, the light treatment that induced calli regeneration differed from the one that caused a higher shoot expression. This fact, along with the quicker response observed in calli cultured in blue light, could indicate that light treatments are highly specific. Thus, it would be suitable to find the best light treatment to acquire pluripotency, another one to proceed to differentiation, and a third one to boost shoot expression.

There is a wide range of responses to light in the different genotypes. In *Prunus*, dark incubation of explants significantly increased the frequency of adventitious shoot regeneration [2,28,29] when compared to a culture in continuous light. Conversely, other authors have found that monochromatic light encourages the multiplication of calli [30], and the combination of different spectra can induce de novo organogenesis [26,27]. In fact, the explants of plant species complete their regeneration process by responding to different photoreceptors under various light spectra [30], and, depending on the genotype, the same spectrum can activate or inhibit the different processes concerning adventitious shoot regeneration [31].

## 4. Materials and Methods

### 4.1. Plant Material

Plant material was obtained from 4-year-old plum (*P. salicina* Lindl) trees grown at the experimental field station of the Instituto Murciano de Investigación y Desarrollo Agrario, Alimentario y Medioambiental (IMIDA) in La Alberca, Murcia (Spain). Nodal segments of the scion cultivar ‘Victoria’ were collected and transferred to the tissue culture laboratory. A total amount of fifty nodal segments were sterilized in a solution of 2% (*v*/*v*) sodium hypochlorite and 0.1% (*v*/*v*) Tween 20 for 2 h. Shoot cultures were established in vitro and subcultured monthly for three months on multiplication medium. Multiplication medium was composed of Murashige and Skoog (MS) salts [32] supplemented with 1 mg L^−1^ of 6-benzyladenine (BA) and 0.1 mg L^−1^ of indolebutyric acid, 3% (*w*/*v*) sucrose, and 0.6% (*w*/*v*) Plant Propagation Agar (Pronadisa, Madrid, Spain). The pH was adjusted to 5.7 with 0.1 N KOH prior to autoclaving at 122 °C (1.1 kg cm^−2^) for 16 min and poured into glass vessels. The proliferating shoots were cultured in climate chambers at 25 ± 1 °C and with a 16 h light period (white LED light [380–780 nm]). After three subcultures, 78 calli were obtained from the base of proliferation clusters. In Figure 1c, organogenic calli can be observed forming spontaneously at the base of the proliferation clusters of *P. salicina* cultivated in vitro. These calli exhibit a compact morphology, with a dense structure and well-defined contours. They have a firm and hard texture with no signs of necrosis or oxidative browning (Figure 5c). The formation of these calli is a common response in *Prunus* species under intensive multiplication conditions. In this case, they appeared with a high frequency, being present in all of the proliferating clusters (Figure 1a,b).

### 4.2. Seed Material

A total of 62 seeds from well-developed fruits of plum cv. ‘Victoria’ were used in the experiment. Fruits were collected from 5-year-old trees grown at the experimental field station of the IMIDA in Calasparra, Murcia (Spain). Fruit flesh was removed, and endocarps containing seeds were washed with water, cleaned with a 0.05% (*v*/*v*) sodium hypochlorite solution, and rinsed under running tap water for 3–5 min until water ran clear. Endocarps containing seeds were dried and then stored in laboratory paper at 4 °C for 3 months. Stored endocarps were cracked to release the seeds (Figure 5a), which were surface sterilized in a solution of 2% (*v*/*v*) sodium hypochlorite and 0.1% (*v*/*v*) Tween 20 for 1.5 h in a laminar flow hood. After disinfection, seed coats were removed and stirred over sterilized paper in a laminar flow hood (Figure 5b).

### 4.3. Explant Preparation

Organogenic calli were sliced to be used in de novo regeneration experiments as previously reported [7]. Each Petri dish contained two of the obtained calli, sliced into seven explants, which sum to a total of fourteen explants per Petri dish (Figure 1a–c). Regarding the second explant source used, seeds were transversally sliced in 5 sections (Axis 1, Axis 2, Cotyledon 1, Cotyledon 2, and Cotyledon 3), as can be observed in Figure 5c. Embryonic axes were transversely cut into two equal segments: the portion that included the epicotyl and the upper part of the hypocotyl was called Axis 1, and the part that included the lower part of the hypocotyl and the radicle was called Axis 2. Cotyledons were cut into three equal segments: the portion closer to the axis was called Cotyledon 1, and the sections that followed were called Cotyledon 2 and Cotyledon 3.

### 4.4. Culture Conditions and De Novo Regeneration

The sections, both of calli and seeds, were cultured in Petri dishes (Figure 5d) containing MS salts supplemented with 0.5 mg L^−1^ of kinetin, 1, 1.5 or 2 mg L^−1^ of BA, 3% (*w*/*v*) sucrose, and 0.6% (*w*/*v*) Plant Propagation Agar (Pronadisa, Madrid, Spain). The pH was adjusted to 5.7 with 0.1 N KOH prior to autoclaving at 122 °C (1.1 kg cm^−2^) for 16 min. Plates were randomly divided into four independent groups that were cultured in four different light spectra. The control group was cultured in white LED light (control; 380–780 nm), a second group was cultured in 55% red (600–700 nm) + 45% far red light (700–800 nm) (red), a third group was cultured in 100% blue light (blue; 400–500 nm), and the last group was cultured in 42% red, 34% far red, and 24% blue light all together (mixed; 400–800 nm) provided by LED lights (Aralab, Madrid, Spain). Explants were cultured in a climate chamber (Aralab 1200 PLH LED, Madrid, Spain) at 25 ± 1 °C and with a 16 h light period.

### 4.5. Experimental Design and Data Collection

Data were recorded from seeds and calli after 28 days of culture. The number of shoots per callus and seed sections was recorded at the end of the experiment. Thus, the regeneration rate (number of shoots per explant) and the percentage of regenerating explants (explants that induced shoots) were calculated, expressed as frequency of organogenic calli (FOC) and frequency of organogenic seed explants (FOSE). Additionally, the type of explant producing de novo shoots and the type of morphogenesis (direct and indirect) were recorded for seeds.

Data were first tested for homogeneity of variance and normality of distribution. Significance was determined with an analysis of variance, and Duncan’s multiple range test at *p* ≤ 0.05 was performed for means separation using Statgraphics18 software (Statgraphics Technologies Inc., Edwardsville, VA, USA).

## 5. Conclusions

Light influence on shoot regeneration in *P. salicina* represents a complex interaction that requires comprehensive investigation due to its variability depending on the explant source and the plant growth regulator doses that are applied to the culture media. This study shows de novo regeneration in ‘Victoria’ through calli obtained from the base of proliferation clusters, which turned out to be more efficient than those obtained from juvenile material. Calli showed a higher efficiency in de novo regeneration compared to seed explants, and both types of explants reacted differently to light and BA treatments. Seed explants were more sensitive to monochromatic light, whereas calli showed a better response to the broader spectrum. BA concentration did not significantly affect somatic shoot induction in any of the explant types. In contrast, a strong interaction between light and BA was observed. While studies on light spectrum continue to evolve, this protocol provides a reliable method for cell editing and transformation.

## Figures and Tables

**Table 1:** Frequency of organogenic callus (FOC) formation in ‘Victoria’ plum calli.

FOC (%)																
BA (mg mL^−1^)	Light															
	Blue				Red				Mix				Control			
**1.5**	76.30	±	11.37	ab	100.00	±	0.00	a	83.33	±	11.67	ab	81.26	±	11.67	Ab
**2**	51.20	±	17.36	b	100.00	±	0.00	a	76.67	±	15.82	ab	83.33	±	16.67	Ab
**2.5**	53.00	±	13.90	b	100.00	±	0.00	a	66.67	±	16.08	ab	85.20	±	3.45	Ab

Values are means ± SE. Letters indicate differences (*p* < 0.05) between treatments.

**Table 2:** Frequency of organogenic seed explant (FOSE) formation in ‘Victoria’ plum seeds.

FOSE (%)																
BA (mg mL^−1^)	Light															
	Blue				Red				Mix				Control			
**1.5**	0.00	±	0.00	d	20.00	±	12.00	d	20.00	±	12.00	bc	33.33	±	13.30	bc
**2**	80.00	±	20.00	ab	40.00	±	14.50	bc	25.00	±	8.00	d	50.00	±	28.90	abc
**2.5**	66.67	±	21.10	abc	80.00	±	20.00	ab	0.00	±	0.00	d	0.00	±	0.00	d

Values are means ± SE. Letters indicate differences (*p* < 0.05) between treatments.

## Data Availability

Data are available upon request.
